# Supplementary material for: Prolonged 3.5 GHz and 24 GHz RF-EMF Exposure Alters Testicular Immune Balance, Apoptotic Gene Expression, and Sperm Function in Rats
Source: Biomedicines. 2025 Oct 11;13(10):2471. doi: 10.3390/biomedicines13102471 (PMC12562081; doi:10.3390/biomedicines13102471)
Supplement: Supplementary file 1 [file biomedicines-13-02471-s001.zip › biomedicines-3898437-supplementary.pdf]

Supplementary Table S1 Testicular cytokines (IL-10, IL-6, IL-1β, TNF-α) across exposure groups. One-way ANOVA with Tukey post-hoc.

Cytokine	Duration	ANOVA F (df)	*p*	Partial η^2 (Post-hoc power)	Post hoc comparison	Mean diff	Adj. p	Cohen's d	Interpretation
**IL-10**	1-hour	13.64 (2,14)	0.0005	0.66	Ctrl vs 3.5 GHz	0.23	0.885	0.28	NS, small effect
					Ctrl vs 24 GHz	2.39	0.0008	2.92	Significant, very large effect
					3.5 vs 24 GHz	2.17	0.0018	2.64	Significant, very large effect
**IL-10**	7-hour	5.44 (2,14)	0.018	0.44	Ctrl vs 3.5 GHz	1.38	0.052	1.56	Borderline NS, very large effect (underpowered)
					Ctrl vs 24 GHz	1.53	0.023	1.75	Significant, very large effect
					3.5 vs 24 GHz	0.16	0.953	0.18	NS, negligible effect
**IL-6**	1-hour	0.35 (2,15)	0.71	0.045	Ctrl vs 3.5 GHz	−0.39	0.953	0.17	NS, negligible effect
					Ctrl vs 24 GHz	−1.10	0.693	0.48	NS, small–medium effect (trend)
					3.5 vs 24 GHz	−0.71	0.857	0.31	NS, small effect
**IL-6**	7-hour	1.77 (2,13)	0.208	0.21	Ctrl vs 3.5 GHz	2.9	0.238	1.04	NS, large effect (underpowered)
					Ctrl vs 24 GHz	0.14	0.996	0.05	NS, negligible effect
					3.5 vs 24 GHz	−2.77	0.296	0.99	NS, large effect (underpowered)
**IL-1β**	1-hour	1.78 (2,15)	0.203	0.19	Ctrl vs 3.5 GHz	−0.36	0.533	0.63	NS, medium effect (underpowered)
					Ctrl vs 24 GHz	−0.63	0.179	1.08	NS, large effect (underpowered)
					3.5 vs 24 GHz	−0.26	0.718	0.45	NS, small–medium effect
**IL-1β**	7-hour	0.073 (2,14)	0.93	0.01	Ctrl vs 3.5 GHz	−0.09	0.994	0.06	NS, negligible effect
					Ctrl vs 24 GHz	−0.34	0.926	0.23	NS, small (trivial) effect
					3.5 vs 24 GHz	−0.24	0.96	0.16	NS, negligible effect
**TNF-α**	1-hour	0.98 (2,15)	0.398	0.12	Ctrl vs 3.5 GHz	−4.29	0.466	0.7	NS, medium effect (underpowered)
					Ctrl vs 24 GHz	0.03	>0.999	0.01	NS, negligible effect
					3.5 vs 24 GHz	4.31	0.462	0.7	NS, medium effect (underpowered)
**TNF-α**	7-hour	11.79 (2,11)	0.0018	0.68	Ctrl vs 3.5 GHz	1.66	0.621	0.67	NS, medium effect (underpowered)
					Ctrl vs 24 GHz	7.18	0.0024	2.91	Significant, very large effect
					3.5 vs 24 GHz	5.52	0.0135	2.23	Significant, very large effect

Supplementary Table S2 Apoptosis-related gene expression (*Casp3, Tp53, Bcl2, Bax*) across exposure groups. One-way ANOVA with Tukey post-hoc.

Gene	Duration	ANOVA F (df)	*p*	Partial η² (Post-hoc power)	Post hoc comparison	Mean diff	Adj. p	Cohen's d	Interpretation
Casp3	1-hour	11.54 (2,15)	0.0009	0.61	Ctrl vs 3.5 GHz	−0.70	0.04	1.56	Significant, very large effect
					Ctrl vs 24 GHz	0.53	0.129	1.2	NS, large effect → underpowered
					3.5 vs 24 GHz	1.23	0.0007	2.76	Significant, extremely large effect
Casp3	7-hour	5.97 (2,14)	0.013	0.46	Ctrl vs 3.5 GHz	−0.14	0.568	0.63	NS, medium effect → underpowered
					Ctrl vs 24 GHz	0.3	0.106	1.33	NS, large effect → underpowered
					3.5 vs 24 GHz	0.44	0.012	1.96	Significant, very large effect
Tp53	1-hour	10.78 (2,14)	0.0015	0.61	Ctrl vs 3.5 GHz	0.53	0.0013	2.73	Significant, extremely large effect
					Ctrl vs 24 GHz	0.18	0.289	0.95	NS, large effect → underpowered
					3.5 vs 24 GHz	−0.34	0.0205	1.78	Significant, very large effect
Tp53	7-hour	0.78 (2,12)	0.48	0.12	Ctrl vs 3.5 GHz	−0.14	0.748	0.49	NS, small–medium effect
					Ctrl vs 24 GHz	0.08	0.917	0.26	NS, small effect
					3.5 vs 24 GHz	0.22	0.453	0.75	NS, medium effect (underpowered)
Bcl2	1-hour	5.39 (2,15)	0.017	0.42	Ctrl vs 3.5 GHz	−0.87	0.061	1.44	NS (borderline), very large effect → underpowered
					Ctrl vs 24 GHz	0.21	0.822	0.35	NS, small effect
					3.5 vs 24 GHz	1.08	0.019	1.79	Significant, very large effect
Bcl2	7-hour	2.83 (2,13)	0.096	0.3	Ctrl vs 3.5 GHz	0.18	0.247	1.09	NS, large effect → underpowered
					Ctrl vs 24 GHz	−0.04	0.942	0.22	NS, small effect
					3.5 vs 24 GHz	−0.21	0.099	1.31	NS (borderline), large effect → underpowered
Bax	1-hour	1.38 (2,14)	0.284	0.17	Ctrl vs 3.5 GHz	−0.16	0.742	0.45	NS, small–medium effect
					Ctrl vs 24 GHz	0.18	0.685	0.51	NS, medium effect
					3.5 vs 24 GHz	0.33	0.254	0.96	NS, large effect → underpowered
Bax	7-hour	1.50 (2,15)	0.255	0.17	Ctrl vs 3.5 GHz	0.23	0.806	0.36	NS, small effect
					Ctrl vs 24 GHz	0.61	0.234	0.99	NS, large effect → underpowered
					3.5 vs 24 GHz	0.39	0.54	0.62	NS, medium effect

Supplementary Table S3 Sperm quality parameters (concentration, motility, viability) across exposure groups. One-way ANOVA with Tukey post-hoc.

Sperm quality	Duration	ANOVA F (df)	*p*	Partial η² (Post-hoc power)	Post hoc comparison	Mean diff	Adj. p	Cohen's d	Interpretation
**Concentration**	1-hour	0.53 (2,14)	0.6	0.07	Ctrl vs 3.5 GHz	0.97	0.987	0.09	NS, negligible effect
					Ctrl vs 24 GHz	5.83	0.608	0.56	NS, medium effect → underpowered
					3.5 vs 24 GHz	4.87	0.727	0.47	NS, small–medium effect
**Concentration**	7-hour	19.93 (2,15)	<0.0001	0.73	Ctrl vs 3.5 GHz	−7.00	0.878	0.28	NS, small effect
					Ctrl vs 24 GHz	74.67	0.0003	3.01	Significant, extremely large effect
					3.5 vs 24 GHz	81.67	0.0001	3.29	Significant, extremely large effect
**Motility**	1-hour	14.68 (2,15)	0.0003	0.66	Ctrl vs 3.5 GHz	30.33	0.0002	3.09	Significant, extremely large effect
					Ctrl vs 24 GHz	11.33	0.146	1.16	NS, large effect → underpowered
					3.5 vs 24 GHz	−19.00	0.011	1.94	Significant, very large effect
**Motility**	7-hour	6.05 (2,15)	0.012	0.45	Ctrl vs 3.5 GHz	23.17	0.009	2.01	Significant, very large effect
					Ctrl vs 24 GHz	11.33	0.237	0.98	NS, large effect → underpowered
					3.5 vs 24 GHz	−11.83	0.211	1.02	NS, large effect → underpowered
**Viability**	1-hour	125.6 (2,15)	<0.0001	0.94	Ctrl vs 3.5 GHz	−0.33	0.992	0.07	NS, negligible effect
					Ctrl vs 24 GHz	37	<0.0001	7.89	Significant, extremely large effect
					3.5 vs 24 GHz	37.3	<0.0001	7.96	Significant, extremely large effect
**Viability**	7-hour	40.87 (2,15)	<0.0001	0.84	Ctrl vs 3.5 GHz	57.67	<0.0001	5.11	Significant, extremely large effect
					Ctrl vs 24 GHz	39	<0.0001	3.46	Significant, extremely large effect
					3.5 vs 24 GHz	−18.67	0.03	1.66	Significant, very large effect
